# Gordon Parker: Staging the bipolar disorders: Are early stages at too early a stage for intervention?

**DOI:** 10.1111/bdi.13278

**Published:** 2022-12-01

**Authors:** Ralph Kupka, Manon Hillegers, Eline Regeer

**Affiliations:** ^1^ Department of Psychiatry Amsterdam University Medical Center Amsterdam The Netherlands; ^2^ Department of Child and Adolescent Psychiatry Erasmus Medical Center Rotterdam The Netherlands; ^3^ Altrecht Center for Mental Health Care Utrecht The Netherlands

In his review, professor Parker critically addresses staging bipolar disorder (BD), especially defining early stages before the onset of a first manic episode, and early intervention for individuals that are at risk to develop BD.^1^ His major points are (1) that subsyndromal signs and symptoms, that in hindsight have preceded clinically manifest BD, especially in persons with a positive family history of BD, are too non‐specific to be prospectively useful as reliable risk factors for impending BD; (2) most individuals “at risk” will not develop BD; and (3) that intervening before the onset of BD has not proven to be effective in the long term, and may even be harmful in various ways by causing concern in parents and offspring or by giving preventive pharmacotherapy. We want to comment on these viewpoints. Early intervention confronts us with the inevitable dilemma to find a balance between detecting the first clinical signs of impending BD and thus avoid delaying effective treatment on the one hand, and labeling a person as being at significant risk for a potential serious and lifelong mental illness at an age when identity is being formed and mood instability is often part of normal emotional development on the other. Clinical staging models, despite all still unresolved issues, can help to make such decisions. Staging serious mental conditions such as BD is intended to define optimal windows of opportunity and give the most effective treatment at the earliest possible moment in the evolving course of illness, thereby preventing illness progression.^2^ Although early (“at risk,” “prodromal,” or “subsyndromal”) stages in clinical staging models inevitably lack specificity for BD, they make clinicians and parents aware that there is a substantial risk of developing an affective (bipolar, depressive, and anxiety) disorder in offspring of individuals with BD.^4^ Moreover, anxiety disorders and depression not rarely make a transition to BD at a later (st)age.^2^, ^3^ Educating these families about known risks and stimulating watchfulness for signs and symptoms that may need further evaluation is effective within the framework of preventive programs.^4^ Parker's point that there is still no evidence that early intervention will prevent BD in the long term may be true, but even if such programs would only delay illness onset or mitigate its manifestations, this is extremely important especially in adolescence and early adulthood. Specific symptomatic treatment, and especially pharmacotherapy, should only be given for clinically manifest psychopathology, such as sleep disorder, anxiety, or depression. Predictive signs (such as anxiety and sleeping problems in childhood, and mood lability or subsyndromal manic symptoms) that have been reported in the studies mentioned by Parker become more meaningful and of clinical relevance when individuals with a positive family history for BDs present with depression. Then there is a need to weight the risk of conversion to BD and to be careful when considering antidepressants. Introducing “preventive” moodstabilizing medication without manifest BD is not recommended and will even be harmful since it inevitably leads to the dilemma how long to continue and when to stop; if no symptoms occur, this may be mistaken as the result of an “effective” intervention. We agree with Parker that we must not scare and stigmatize families by predicting serious mental illness in an oversimplified context. Still, while facing the facts with nuance, we have to be clear about the increased risk for mood disorders in offspring of individuals with BD and depression, and take timely and appropriate action.^5^

## CONFLICT OF INTEREST

The authors (RK, ER, and MH) have no conflict of interest with regard to this paper.

## Data Availability

Data sharing is not applicable to this article as no new data were created or analyzed in this study.
